# Systematization of analytical studies of polycythemia vera, essential thrombocythemia and primary myelofibrosis, and a meta-analysis of the frequency of *JAK2*, *CALR* and *MPL* mutations: 2000–2018

**DOI:** 10.1186/s12885-019-5764-4

**Published:** 2019-06-17

**Authors:** Mónica Mejía-Ochoa, Paola Andrea Acevedo Toro, Jaiberth Antonio Cardona-Arias

**Affiliations:** 1 Molecular Hematopathology Research Group, School of Microbiology,University of Antioquia, Laboratorio Médico de referencia, Medellin, Colombia; 2 Molecular Hematopathology Research Group, School of Microbiology, University of Antioquia, Medellin, Colombia; 3 School of Microbiology University of Antioquia, School of Medicine, Cooperativa Universidad de Colombia, Calle 67 Número 53 – 108, Bloque 5, oficina 103, Medellin, Colombia

**Keywords:** Myeloproliferative disorders, Mutation, Polycythemia vera, Essential thrombocythemia, Primary myelofibrosis, Meta-analysis

## Abstract

**Background:**

Research into Philadelphia-negative chronic myeloproliferative neoplasms is heterogeneous. In addition, no systematization of studies of polycythemia vera (PV), essential thrombocythemia (ET) or primary myelofibrosis (PMF) have been carried out. The objective of this review is to characterize studies on *BCR-ABL1*-negative chronic myeloproliferative neoplasms and to compare the frequency of *JAK2, MPL* and *CALR* mutations in PV, ET and PMF.

**Method:**

A systematic review of the scientific literature was conducted, as was meta-analysis with an ex-ante selection of protocol, according to phases of the PRISMA guide in three interdisciplinary databases. To guarantee reproducibility in the pursuit and retrieval of information, the reproducibility and methodological quality of the studies were evaluated by two researchers.

**Results:**

Fifty-two studies were included, the majority having been carried out in the United States, China, Brazil and Europe. The frequency of the *JAK2V617F* mutation ranged from 46.7 to 100% in patients with PV, from 31.3 to 72.1% in patients with ET, and from 25.0 to 85.7% in those with PMF. The frequency of the *MPL* mutation was 0% in PV, from 0.9 to 12.5% in ET, and from 0 to 17.1% in PMF. The *CALR* mutation occurred at a frequency of 0.0% in PV, whereas in ET, it ranged from 12.6 to 50%, and in PMF, it ranged from 10 to 100%. The risk of this mutation presenting in PV is 3.0 times that found for ET and 4.0 times that found for PMF.

**Conclusion:**

Given the specificity and reported high frequencies of the *JAK2V617F, MPL* and *CALR* mutations in this group of neoplasms, the diagnosis of these diseases should not be made on clinical and hematological characteristics alone but should include genetic screening of patients.

## Background

Myeloproliferative neoplasms (MPN) is a group of eight clinical entities that include those of the *BCR-ABL1*-negative phenotype, named Philadelphia-negative MPN. These diseases are generated by a clonal disorder in hematopoietic stem cells that leads to an excessive production of mature cells and their accumulation in peripheral blood. The affected lineages of these disorders are erythroid, megakaryocytic and granulocytic, which generate polycythemia vera (PV), essential thrombocythemia (ET) and primary myelofibrosis (PMF). PMF, according to the most recent World Health Organization (WHO) classification of 2016, is subdivided into profibrotic and fibrotic states [1].

From a clinical point of view, patients with MPN are at risk of presenting vascular complications including venous or arterial thrombosis and bleeding. On the other hand, patients with PV and ET can progress to PMF, and all patients with MPN may develop an acute phase of myeloid leukemia [2].

The incidence of PV per 100,000 inhabitants is 0.84 at the global level, and in Europe and North America it has been registered at 0.86 and 0.74 respectively. The data for ET includes an incidence of 1.03 in the world, 1.03 in Europe and 0.96 per 100,000 in North America. For PMF, a global incidence of 0.47 per 100,000 inhabitants has been reported, which does not show marked variation among regions. It is necessary to mention that many studies have been carried out in high-income countries, so it is probable that in middle-income and low-income countries, MPN is misclassified and the incidences may be underestimated [3]. For example, the incidence of MPN in Colombia is not known. However, according to statistics of the National Cancer Institute, of 251 hematological neoplasms diagnosed in 2011, only eight were from the MPN group [4, 5].

In 1951, William Dameshek was the first to propose that MPN, mainly chronic myeloid leukemia (CML), PV, ET and PMF, should be categorized as a single group because patients share clinical and laboratory characteristics such as insidious onset, hepatomegaly, splenomegaly and increased cellularity in bone marrow [6]. This led to the diagnosis, risk stratification, clinical characterization and evaluation of the prognosis of patients with MPN being based on hematological analysis of bone marrow and peripheral blood. However, since 2005, with the advent of molecular biology, this panorama changed notably after the discovery of mutations in *JAK2 (Janus kinase 2)*, *MPL (trombopoietin receptor)* and *CALR (calrreticulin)* genes identified in PV, ET and PMF. The presence of these markers and the absence of other genetic markers such as *BCR-ABL1* have clarified the pathogenesis of these diseases [7], indicating the heterogeneity of the bases of these neoplasms while allowing them to be reunited in the subgroup of *BCR-ABL*1-negative Chronic MPN as an independent group of chronic myeloid leukemia [8–10].

With this in mind, multiple investigations have been conducted on this group, some of which aimed to determine the frequency and utility of these mutations in the diagnosis, clinical monitoring and prognosis of MPN, demonstrating how their presence, order of acquisition and association with other molecular markers play important roles in the onset of disease [11]. Other studies at the genomic level have focused on determining the frequency and clinical and prognostic implications of *JAK2*, *CALR* and *MPL* mutations called “drivers” [12, 13]. The prevalence of these markers is variable among the studies; for example, in PV, *JAK2* frequencies from 46.7 to 100% have been reported, and the frequency of *CALR* in ET also varies, with reports from 12.6 to 50%. Other studies have focused on additional markers related to cellular signaling pathways (tyrosine kinase), oxidative stress, cell cycle (p53) and epigenetic events [14–17]. Likewise, in the evaluation of prognostic factors and complications, different clinical variables have been measured, such as the parameters of blood count, types of treatments used and the patients’ age, among others [18, 19].

Currently there are different research approaches to address this group of diseases, which allows the evaluation of a large number of variables to compare various characteristics of patients with PV, ET and PMF. With the diversity of studies, some systematic reviews have focused on molecular issues such as the relationship of a specific mutation with clinical characteristics or prognosis of a single disease [20, 21], the comparison of two types of mutations at the prognostic level [22, 23], diagnosis and therapy related to a single mutation [24] and factors associated with the lifestyle, environmental, ethnic and family conditions of patients [25].

Despite this background, no study has characterized the publications made in this subgroup of neoplasms, particularly regarding variables such as years of publication, countries, study populations, detection methods and main study objectives. Additionally, it is necessary to systematize analytical studies comparing different aspects of PV, ET and PMF given that, despite their similarities, there are genetic and clinical markers that differentiate this subgroup, among which is frequency of mutations such as *JAK2*, *MPL* and *CALR*. Additionally, given the large number of reports on these frequencies, it is necessary to obtain a global measure that summarizes the large number of publications and allows the real prevalence of genetic markers in the subgroup of *BCR-ABL*-negative MPN to be known. In the scientific literature, there is no systematic review available of analytical studies about the frequency of these markers. In addition to being major diagnostic criteria, these studies provide relevant information on the survival, risk of thrombosis and stratification of patients with favorable or unfavorable prognoses [10].

Therefore, the objective of this review is to characterize the studies of *BCR-ABL1*-negative chronic myeloproliferative neoplasms and to compare the frequency of *JAK2*, *MPL* and *CALR* mutations in PV, ET and PMF.

## Methods

### Type of study

A systematic review of scientific literature with meta-analysis.

### Research protocol according to the PRISMA guide

Preferred reporting items for systematic reviews and meta-analyses [26]

#### Identification

A sensitivity search was carried out (without circumscribing the terms of the thesauri, particularly DeCS and MeSH), on three interdisciplinary databases: Medline-Pubmed, Scielo, and Science Direct. Additionally, a manual search in Google Scholar was conducted, in which no studies were found in addition to those identified in the aforementioned three databases. These three databases guarantee the completeness of the protocol, given that Pubmed is an interdisciplinary database of the National Library of Medicine of the United States that has accumulated more than 14 million references for biomedical articles since 1950. Scielo is equally interdisciplinary and collects scientific publications from the Spanish and Latin American community, and Science Direct is one of the largest electronic collections in the world.

The identification of the search terms was done by a “*pearl harvest*”, combining the stages of the traditional method and the exhaustive method; that is, to identify relevant articles on the subject, preferably from bibliographic reviews; find the key terms for indexing; search other relevant articles in the database with these terms; and determine other key databases for the search. This process was performed for each database until no new articles were found. The following terms were selected: *BCR-ABL1* negative, Chromosome Ph, Philadelphia chromosome, Philadelphia -Ph- chromosome and Philadelphia translocation [27]. These terms correspond to those frequently used by the experts in the area to describe our study object, which is BCR-ABL1-negative MPN. Likewise, the use of these terms excludes BCR-ABL1-positive neoplasms from the search, which are mainly chronic myeloid leukemia. With each term, an independent search was made combining all terms with the Boolean operator ‘or’; for example, [(*BCR-ABL* Negative) OR ((Chromosome Ph) OR (Philadelphia chromosome) OR (Philadelphia -Ph- chromosome) OR (Philadelphia translocation))]. This step was complemented with a manual search in the references of the selected articles and other manuscripts recommended by experts.

#### Screening and application of inclusion criteria

Studies were included that had the search terms in the title, abstract or keywords; lacked time or language limits; were original articles, excluding narrative reviews, short communications, letters to the editor and books; had a main theme of the *BCR-ABL1* negative MPN; and described research conducted in humans and in vivo studies. The obtained articles were exported to a common source (EndnoteWeb) and duplicate papers were eliminated.

Some of the syntax used were chromosome ph [Title/ Abstract]; *BCR-ABL* Negative [Title / Abstract]; TITLE-ABSTR-KEY (*BCR ABL* Negative); ((((*BCR ABL* Negative [Title/Abstract]) OR Chromosome Ph [Title / Abstract]) OR Philadelphia chromosome [Title / Abstract]) OR Philadelphia (Ph) chromosome [Title / Abstract]) OR Philadelphia translocation [Title/Abstract]]; TITLE-ABSTR-KEY (*BCR ABL* Negative) or TITLE-ABSTR-KEY (Chromosome Ph OR Philadelphia chromosome OR Ph chromosome OR Philadelphia translocation); (ti: ((ab: (*BCR ABL* Negative OR Chromosome Ph OR Philadelphia chromosome OR Ph chromosome OR Philadelphia translocation)))).

This protocol did not present temporal limitations retrospectively, and prospectively, a final update was made in February of 2018. The delimitation of the temporality of the title was based on the decade of the oldest study included in the review.

#### Selection and application of the exclusion criteria

In this phase, the following articles were excluded: those with a number of patients less than 10 because these corresponded to case studies or series of cases, manuscripts that were not available in the databases and no response from the authors was received, studies with incomplete data in central variables of the study (such as those that did not specify the number of patients or the type of diagnosis), descriptive studies that evaluated only one disease of the group, experimental or clinical studies and studies that evaluated diagnostic tests.

#### Inclusion

The studies that met the above criteria were included in the review. Variables for title, authors, type of study, main subject of the study, journal, year of publication, first author, country of study, number of total patients and by type of disease, demographic and clinical characteristics of the patients, and the main objective of the study were extracted from the manuscripts. With the studies that reported the frequency of mutations, a quantitative synthesis was performed.

### Evaluation of reproducibility and methodological quality

The application of the search and selection protocol of the studies was carried out by two researchers independently to guarantee the reproducibility of the review; a priori, it was determined that the discrepancies would be resolved by consensus. The variable extraction phase was carried out independently by two researchers, and a Kappa index of 1.00 was obtained for the variables of country, year, population, and mutation record. Other variables (since their report was text) were made reproducible by a third reviewer. To evaluate the methodological quality, the STROBE (Strengthening the Reporting of Observational studies in Epidemiology) guide was applied, which includes criteria for evaluating the internal and external validity of the included studies [28]. For each article, the compliance with the 22 criteria of the STROBE guide was determined.

### Analysis of the information

The variables were described via frequencies. The frequency of the *JAK2V617F* mutation was compared in the study diseases (PV, ET and PML) with a meta-analysis of odds ratios with eight studies that used AS-PCR (Allele-specific polymerase chain reaction) as detection technique because the other techniques were represented by few studies or the methodology of sequencing was not fully explained. For the MPL and CALR mutations, the use of the AS-PCR was less frequent, so a meta-analysis was explored for sequencing and fragment analysis. In all meta-analyses, heterogeneity was assessed with Galbraith, Dersimonian and Laird (Q statistic with Chi-square distribution) and RI coefficient, the publication bias with Begg statistic and funnel plot, and the sensitivity analysis with an influences graph. The final results of the meta-analysis are presented as forest plots. Subsequently, a meta-regression was performed by diagnostic criteria for *JAK2*, separating articles that used WHO criteria from 2001 and 2008.

## Results

**Fig. 1:**
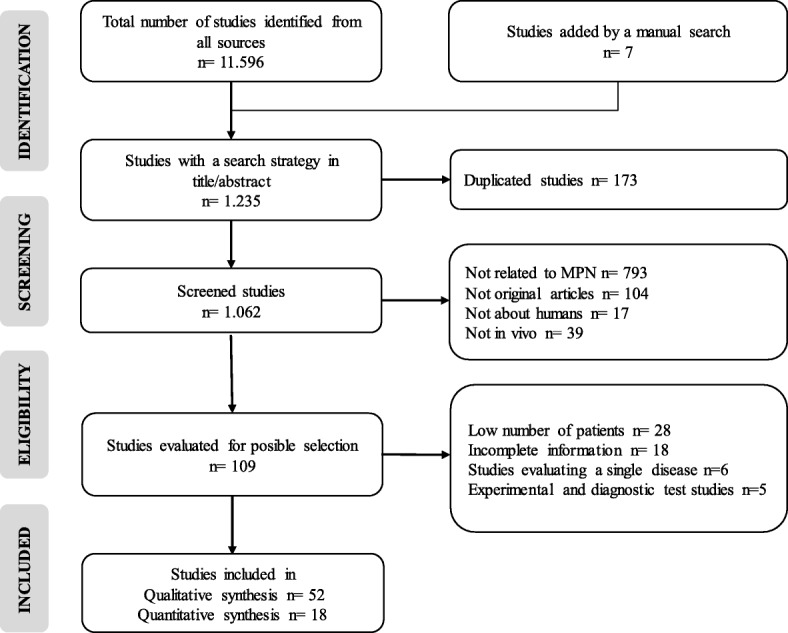
Flow-gram of search and selection of articles

In the application of the search and selection protocol, 11,596 studies were obtained without the application of limits or filters to the databases. This number was reduced to 1228 that included the terms in title or abstract, to these were added 7 from the manual search. After the application of the inclusion and exclusion criteria, a qualitative synthesis of the information of 52 articles and a quantitative synthesis (meta-analysis) of 18 studies that compared the frequency of the *JAK2*V617F, *MPL* and *CALR* mutation by type of disease were performed (Fig. 1).

**Fig. 2:**
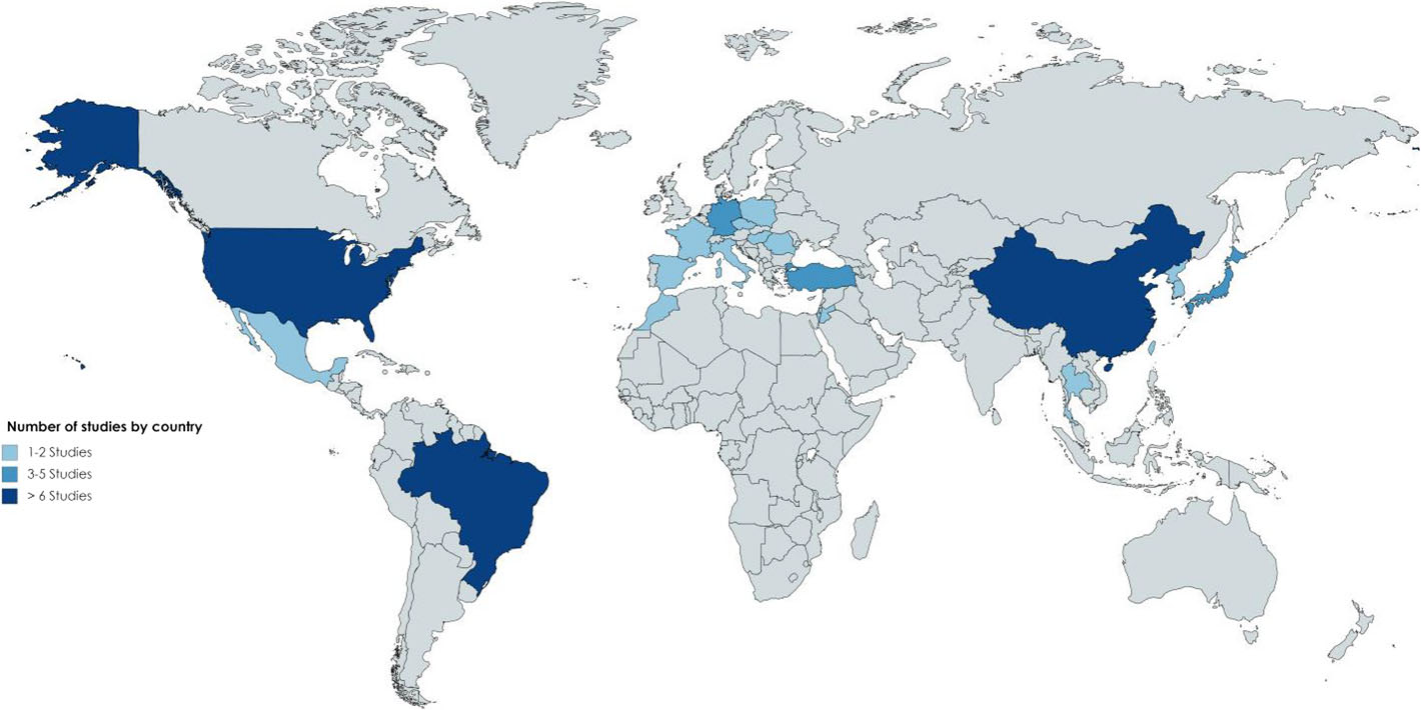
Absolute frequency of studies by country

The selected studies were published in 20 different countries. The United States, China, and Brazil had the highest number of studies with 10, nine and six, respectively. The other countries had between one and three publications (Fig. 2).

**Fig. 3:**
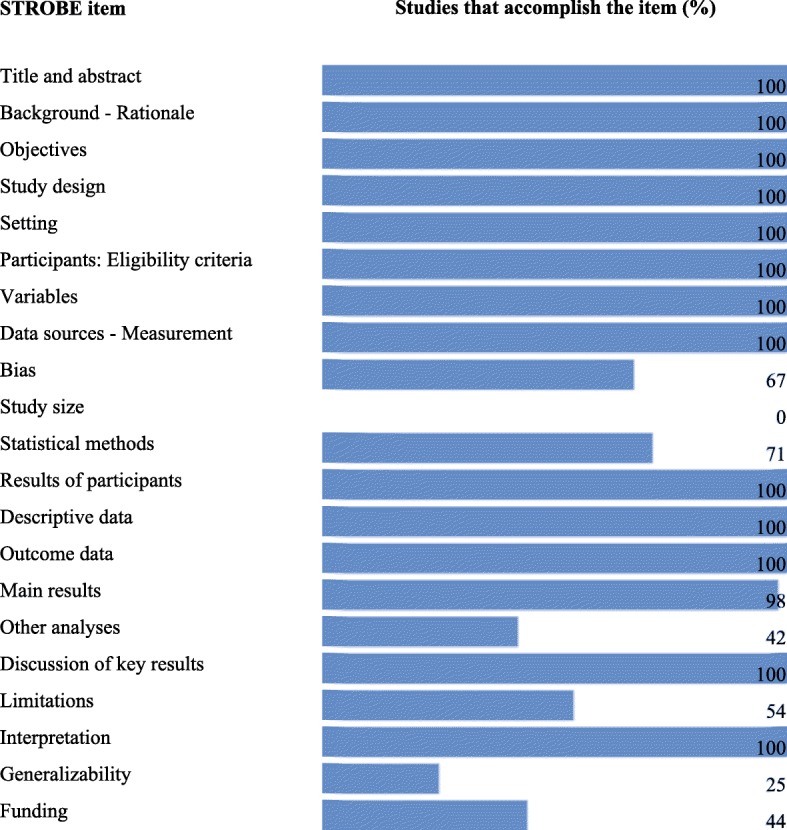
Evaluation of methodological quality

An evaluation of the methodological quality of the included studies showed that the percentage of compliance was greater than 70% of the criteria of the STROBE guide. The criteria that had the lowest compliance percentage were the explanation of the sample size and the discussion of the possibility of generalizing the results obtained (Fig. 3).

Studies published between 2003 and 2018 were identified. The highest proportion were in 2016, with 19.2% (*n* = 10 studies), and 2015, with 17.3% (*n* = 9 studies), whereas between 2003 and 2014, the frequency of studies ranged between one and four per year. A total of 27,078 study subjects were included, of which 12.9% (*n* = 3488) corresponded to patients with PV, 19.6% (*n* = 5300) with ET, 7.2% (*n* = 1954) with PMF and 60.3% to healthy controls (*n* = 1070) and people with other diseases (*n* = 15,266) (Table 1).

**Table 1:** Description of the studies included in the review according to the year of publication and the number of subjects studied

Author	Year	Country	N PV	N ET	N PMF	N others
Aviram A [17]	2003	Israel	31	56	10	0
Hsu H [29]	2004	Taiwan	16	30	0	17^a^51^b^
Jelinek J [30]	2005	U.S.A.	29	10	19	316^a^
Jost E [31]	2007	Germany	9	7	0	23^a^
Zhang S [32]	2007	China	23	40	8	23^a^
Najfeld V [33]	2007	U.S.A.	18	0	6	15^a^
Suzuki R [34]	2007	Japan	15	21	5	8^a^
Xu W [35]	2008	China	32	102	13	43^a^50^b^
Lucia E [36]	2008	Italy	108	74	26	25^a^50^b^
Tefferi A [37]	2009	U.S.A.	26	13	32	0
Tefferi A [38]	2009	U.S.A.	89	57	60	33^a^
Aranaz P [14]	2010	Spain	4	15	4	21^a^
Tripodi J [39]	2010	U.S.A.	47	21	52	26^a^
Chen X [40]	2011	China	15	70	18	0
Toyama K [41]	2011	Japan	25	82	8	0
Benmoussa A [42]	2011	Morocco	19	8	12	31^a^
da Silva R [43]	2012	Brazil	52	81	11	0
Zhang X [44]	2012	China	51	66	17	0
Ho C [45]	2012	Taiwan	21	32	5	20^a^
Schnittger S [46]	2012	Germany	736	615	230	13961^a^
Pagliarini S [47]	2013	Brazil	17	22	12	5^a^90^b^
Patriarca A [48]	2013	Italy	26	55	9	8^a^
Kim H [49]	2013	Korea	26	42	7	3^a^
Wu Z [50]	2014	China	80	80	50	0
Kissova J [18]	2014	Czech Republic	41	105	36	0
Payzin K [51]	2014	Turkey	81	129	22	0
Macedo L [52]	2015	Brazil	38	42	33	30^a^150^b^
Ouyang Y [53]	2015	China	48	171	27	180^a^20^b^
Kander E [19]	2015	U.S.A.	118	144	63	26^a^
Mahjoub S [54]	2015	France	22	17	17	0
Jaradat S [55]	2015	Jordan	27	16	14	0
Labastida N [56]	2015	Mexico	14	8	4	0
Duangnapasatit B [57]	2015	Thailand	68	83	6	0
Geduk A [58]	2015	Turkey	7	43	16	30^b^
Lin Y [59]	2015	China	234	428	187	80^a^
Berzoti M [60]	2016	Brazil	14	24	9	60^a^35^b^
Macedo L [61]	2016	Brazil	33	35	22	33^a^123^b^
Didone A [62]	2016	Brazil	20	28	20	68^a^
Xu J [63]	2016	China	171	269	188	0
McFarland D [64]	2016	U.S.A.	34	31	31	21^a^
Wang J [65]	2016	U.S.A.	17	11	23	42^a^43^b^
Gardner J [66]	2016	U.S.A.	7	3	3	34^a^
Trifa A [15]	2016	Romania	140	140	48	363^b^
Delic S [67]	2016	Germany	30	40	30	0
Orvain C [68]	2016	France	42	93	35	86^a^ 26^b^
Goel S [69]	2017	U.S.A.	51	19	73	0
Smaili W [70]	2017	Morocco	0	22	11	0
Gadomska G [71]	2017	Poland	19	46	7	39^b^
Yildiz I [72]	2017	Turkey	23	68	9	0
Li M [73]	2017	China	508	1049	91	0
Misawa K [74]	2018	Japan	166	212	88	27^a^
Gángó A [75]	2018	Hungary	0	425	227	0
Total			3488	5300	1954	16,336

^a^Other diseases (hematológicas) ^b^Healthy controls

The results of the studies showed a high degree of heterogeneity. The first group included studies that genetically characterized MPN from different aspects, among which the detection of methylations [17] and epigenetic alterations [31], the search of structural rearrangements in *JAK2* [33], comparative genomics for the diagnosis [37], the identification of new genetic markers [14], and mutations associated with pathogenesis [60] or mutations in *CALR* and *MPL* in negative *JAK2* patients [41, 70] all stood out. Another group of studies focused on clinical aspects of MPN, describing hemorrhagic complications [19], clinical manifestations [57], symptomatology [63] or the relationship of hematological characteristics with clinical complications [18]. Finally, a third subgroup included publications related to the characterization of cellular and metabolic processes, such as the measurement of the expression of the protein B catenin in *BCR-ABL*-negative MPN [58], the relationship of immune modulators with MPN [65], polymorphisms in oxidative stress genes [15] or the measurement of serum levels of proteins related to angiogenesis in MPN [71].

Of the included studies, twenty analyzed the frequency of the *JAK2V617F* mutation in PV, ET and PMF. These show heterogeneous results in the diagnostic techniques and the proportion of patients with the mutation. The proportion of patients with the mutation ranging from 46.7 to 100% in patients with PV, from 31.3 to 72.1% in patients with ET, and from 25.0 to 85.7% in those with PMF (Table 2). Of these studies, eight used the AS-PCR test to detect the mutation. Based on this information, three meta-analyses were performed to compare the frequency of the mutation among the three diseases.

**Table 2:** Absolute and relative frequencies of the *JAK2V617F* mutation

Author	Diagnostic criteria	Technique	PV % (n)	ET % (n)	PMF % (n)
Zhang SJ [32]	No data	AS-PCR	69.6 (16)	45.0 (18)	37.5 (3)
Suzuki R [34]	WHO 2001	Sequencing	46.7 (7)	47.6 (10)	80.0 (4)
Xu W [35]	WHO 2001	AS-PCR	62.5 (20)	42.2 (43)	38.5 (5)
Lucia E [36]	WHO 2001	AS-PCR	90.2 (74)	72.1 (31)	63.2 (12)
Tefferi A [38]	WHO 2001	RT-PCR	89.9 (80)	45.6 (26)	55.0 (33)
Toyama K [41]	WHO 2008	AS-PCR	88.0 (22)	68.3 (56)	75.0 (6)
Benmoussa A [42]	No data	AS-PCR	89.5 (17)	62.5 (5)	33.3 (4)
Zhang X [44]	No data	AS-PCR	84.3 (43)	69.7 (46)	52.9 (9)
Ho C [45]	No data	HRM	76.2 (16)	46.9 (15)	80.0 (4)
da Silva R [43]	No data	PCR-RFLP	88.5 (46)	48.1 (39)	72.7 (8)
Kim H [49]	WHO 2008	AS-PCR	88.5 (23)	57.1 (24)	85.7 (6)
Patriarca A [48]	WHO 2008	Real-time PCR	100 (26)	63.6 (35)	66.7 (6)
Wu Z [50]	WHO 2008	HRM Sequencing	82.5 (66)	56.3 (45)	58.0 (29)
Payzin K [51]	WHO 2008	Real-time PCR	95.1 (77)	68.2 (88)	77.3 (17)
Labastida N [56]	Conventional	ARMS	62.5 (5)	35.7 (5)	25.0 (1)
Mahjoub S [54]	WHO 2008	AS-PCR	72.7 (16)	47.1 (8)	66.7 (2)
Jaradat S [55]	WHO 2001	Sequencing	70.4 (19)	31.3 (5)	14.3 (2)
Gardner J [66]	WHO 2008	Fragment Analysis PCR	100.0 (7)	66.7 (2)	33.3 (1)
Didone A [62]	WHO 2008	PCR-RFLP	95.0 (19)	71.4 (20)	40.0 (8)
Yildiz I [72]	WHO	Sequencing	73.9 (17)	61.8 (42)	55.6 (5)

*WHO* World Health Organization, *AS-PCR* Allele-specific polymerase chain reaction, *RT-PCR* Reverse transcription polymerase chain reaction, *HRM* High-Resolution Melt, *PCR-RFLP* polymerase chain reaction- Restriction Fragment Length Polymorphism, *ARMS* Amplification-refractory mutation system

**Fig. 4:**
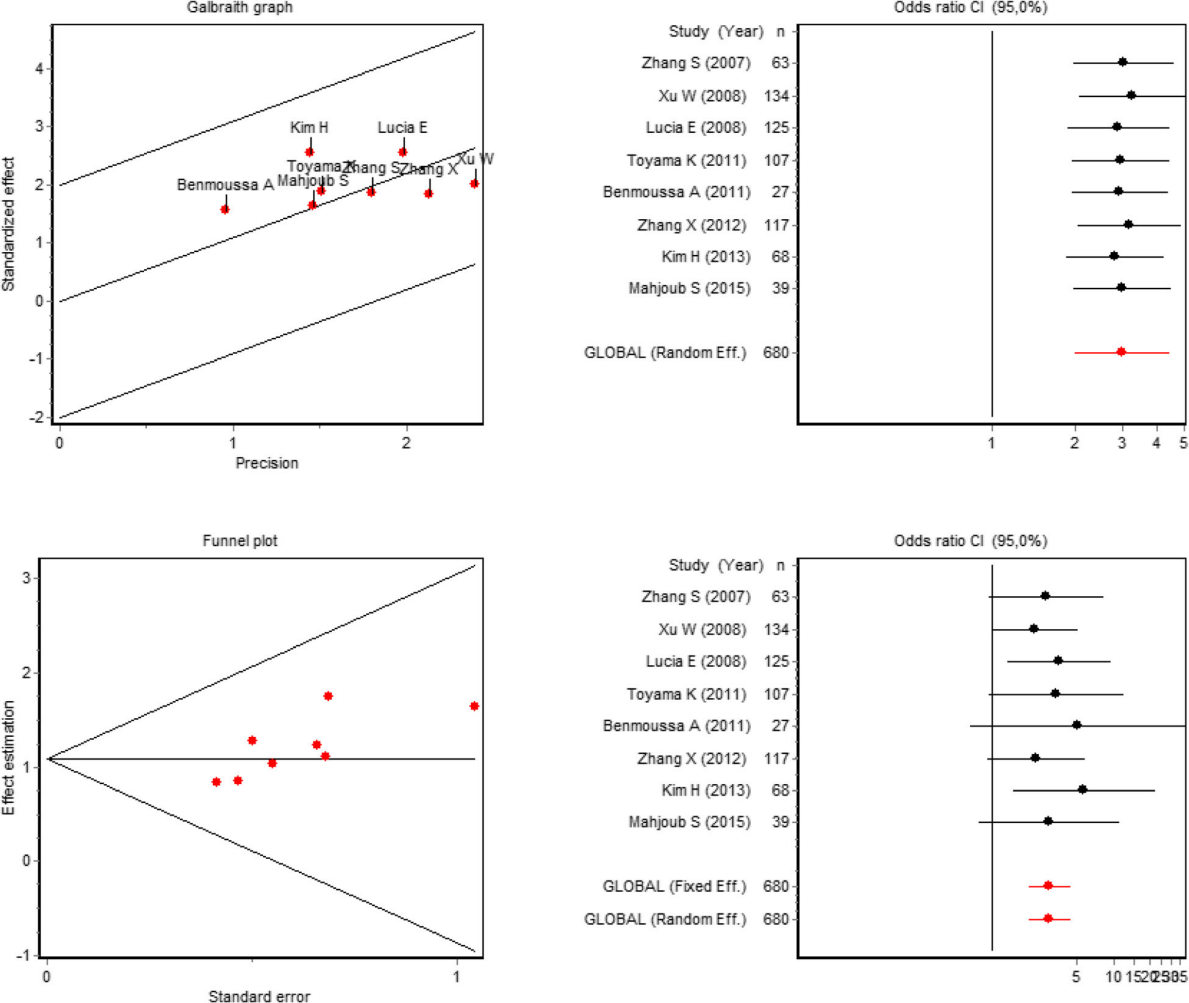
Meta-analysis for the comparison of the frequency of *JAK2* mutations in PV compared to ET

When comparing the odds ratios for the frequency of mutations in patients with PV compared to patients with ET, homogeneity was found between the studies (Galbraith chart). No publication bias was presented according to a funnel plot, and an impact graph confirmed the relevance of a combined measure insofar as the exclusion of each study in successive stages did not generate changes in the summary measure. The accumulated meta-analysis corroborated that the conclusion derived from the summary measure did not change with an increase of sample size or the inclusion of additional patients (Fig. 4).

**Fig. 5:**
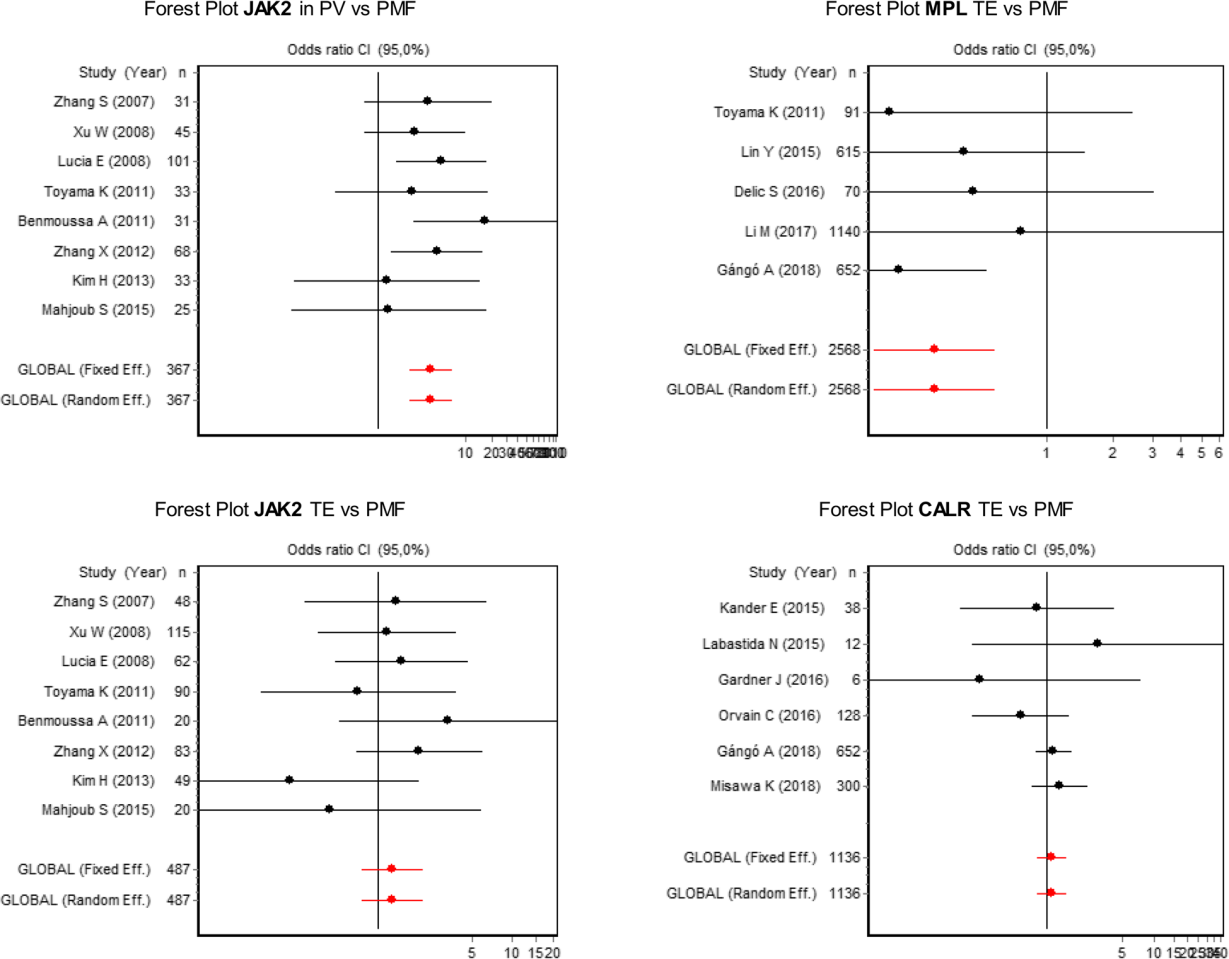
Forest plot for the comparison of the frequency of *JAK2* mutations between PV vs PMF and ET vs PMF, comparison of *MPL* mutations in ET vs PMF and of *CALR* mutations in ET vs PMF

Finally, the odds ratio for the comparison of the frequency of mutations in patients with PV versus subjects with ET, based on a fixed-effect meta-analysis, was 3.0 (95% CI = 2.0–4.4), for the comparison between PV and PMF it was 4.0 (95% CI = 2.3–7.0), whereas in the comparison between ET and PMF an OR of 1.3 was obtained (95% CI = 0.8–2, 2). This indicates that the risk of presenting this mutation in patients with PV is 3.0 times more likely to be found in ET and 4.0 times more frequently observed in people with PMF, whereas the probability of finding the mutation in ET and PMF is statistically similar (Fig. 5).

The MPL mutation was analyzed in 14 studies, and presented with the frequencies 0% in PV, from 0.9 to 12.5% in ET, and from 0 to 17.1% in PMF. The CALR mutation was reported in 13 studies, with a frequency of 0.0% in PV. In ET it ranged from 12.6 to 50% and in PMF from 10 to 100% (Table 3).

**Table 3:** Absolute and relative frequencies of the *MPL* and *CALR* mutations

	MPL Mutation				CALR Mutation			
Author	Technique	PV % (n)	ET % (n)	PMF % (n)	Technique	PV % (n)	ET % (n)	PMF % (n)
Xu [35]	AS-PCR	0 (0)	1.0 (1)	0 (0)	ND	ND	ND	ND
Chen [40]	AS-PCR	0 (0)	1,4 (1)	5,6 (1)	ND	ND	ND	ND
Toyama [41]	Sequencing	0 (0)	2,4 (2)	0 (0)	ND	ND	ND	ND
Wu [49]	Real-time PCR	0 (0)	5,0 (4)	6,0 (3)	Sequencing	0(0)	25,0 (2)0)	20,0 (16)
Labastida [55]	ARMS	0 (0)	12,5 (1)	0 (0)	Fragment analysis	0 (0)	50,0 (4)	25,0 (1)
Trifa [15]	SNP	0 (0)	1,4 (2)	4,2 (2)	SNP	0 (0)	29,3 (41)	25,0 (12)
Gadomska [70]	ND	ND	5,3 (1)	0 (0)	ND	ND	31,6 (6)	100 (2)
Li [72]	Sequencing	ND	0,9 (9)	1,1 (1)	Sequencing	ND	12,6 (132)	12,1 (11)
Schnittger S [46]	PCR	ND	7,1 (44)	13,2 (17)	ND	ND	ND	ND
Misawa [73]	AS-PCR	0 (0)	4,7 (10)	1,1 (1)	Fragment analysis	0 (0)	26,9 (57)	21,6 (19)
Gángó [74]	Sequencing	ND	1,6 (7)	7,1 (16)	Fragment analysis	ND	24,5 (104)	21,6 (49)
Lin [58]	Sequencing	0 (0)	1,2 (5)	2,7 (5)	Sequencing	0 (0)	22,7 (97)	17,6 (33)
Delic [66]	Sequencing	0 (0)	5,0 (2)	10 (3)	Sequencing	0 (0)	25,0 (10)	10,0 (3)
Orvain [67]	Real-time PCR	ND	2,2 (2)	17,1 (6)	Fragment analysis	ND	12,9 (12)	20.0 (7)
Kander [19]	ND	ND	ND	ND	Fragment analysis	ND	33,3 (10)	37,5 (3)
Gardner [65]	ND	ND	ND	ND	Fragment analysis	0 (0)	33,3 (1)	66,7 (2)
Smaili [69]	ND	ND	ND	ND	Sequencing	ND	50,0 (11)	36,4 (4)

*AS-PCR* Allele-specific polymerase chain reaction, *PCR* polymerase chain reaction, *ARMS* Amplification-refractory mutation system, *SNP* single-nucleotide polymorphism, *ND* No Data

In *MPL* and *CALR,* only 3 studies used AS-PCR, so only one combined measure was estimated for sequencing and fragment analysis, without finding heterogeneity between the studies (Galbraith). No publication bias was found, and the sensitivity analysis demonstrated strength in the combined measure.

Based on a meta-analysis under a fixed-effect model, the comparison of the frequency of *MPL* in ET and PMF showed an OR of 0.3 (95% CI = 0.2–0.6), and for *CALR* between ET and PMF, the OR was 1.1 (95% CI = 0.8–1.5). These findings demonstrate that the probability of presenting the *MPL* mutation in PMF is approximately three times greater than for ET, whereas the frequency of *CALR* is statistically similar in both diseases (Fig. 5).

Five studies reported the frequency of the *JAK2* mutation with the diagnostic criteria recommended by the WHO 2001 [34–36, 38, 55]. In their combined measurement, an OR of 3.5 was obtained (95% CI = 1.6–7.7). Eight studies used the WHO 2008 criteria [41, 48–51, 54, 62, 66], for which an OR of 4.7 (95% CI = 3.1–7.5) was found. Three studies used WHO 2008 criteria and AS-PCR, and their combined measurement was an OR of 3.8 (95% CI = 1.6–8.3). These analyzes by subgroups showed that *JAK2* is more frequent in PV than in ET, regardless of the technique or diagnostic criterion used. Although the comparison of the intervals did not show significant differences, greater strength of association was observed when applying the WHO 2008 criteria.

For the frequency of the *CALR* and *MPL* mutations, it was not possible to perform a meta-regression according to the diagnostic criteria, because the subgroups only contained two to three studies.

## Discussion

In this review, 52 studies comparing different variables in 3488 people with PV, 5300 with ET, and 1954 with PMF were systematized, as were 1070 controls, with a majority of the studies being from the United States, China, and Brazil. A total of 20 studies analyzed the frequency of the *JAK2V617F* mutation in the three diseases, and meta-analysis showed that the risk of presenting this mutation in PV is much higher than for ET and PMF. Fourteen studies analyzed the frequency of the MPL mutation, and meta-analysis showed that the probability of having the mutation is higher in PMF than in ET. The frequency of CALR was reported in 13 studies, and no significant differences were found between the two diseases. These findings demonstrate the high external validity of this review while highlighting following factors: the small number of analytical investigations that have been carried out, a high diversity in the comparison of clinical and genetic parameters in Philadelphia-negative MPN, and that few studies of the frequency of mutations exist. These together show a great potential for the orientation and consolidation of research in this group of neoplasms.

It is evident from the publications included in this systematic review that the largest proportion of studies come from the United States, China, and Brazil, which indicates the increasing interest these countries have in studying MPN, as is the case with European countries also. In general, most of the publications come from medium- and high-income countries, which could imply a relationship between the research development in this area and the access or availability of reference centers for the diagnosis and treatment of this type of disease. In the United States, for example, there are the American Society of Hematology, the Leukemia and Lymphoma Society, and the Research Foundation in Myeloproliferative Neoplasms; in Brazil the Brazilian Association of Hematology, Hemotherapy and Cell Therapy; in Europe the European Association of Hematology and the Spanish Society of Hematology and Hemotherapy.

This contrasts with other places with a low frequency of studies. In Colombia, for example, where the incidence of this group of diseases has not been determined, only a few studies describing the clinical characteristics of patients with MPN have been conducted, one of which is a publication by the Colombian Association of Hematology and Oncology. This indicates the myriad obstacles to carrying out this type of research, such as the deficit of professionals in hematopathology and the lack of access to specialized studies for diagnosis [5].

In this systematic review, ET was the most frequently reported disease, with a total of 5300 cases corresponding to 49.34% of all patients with MPN, followed by PV with 32.47% and PMF with 18,19%. This distribution correlates with the incidents reported worldwide for this group of neoplasms. For example, a recent study conducted in Korea, with 4342 patients showed higher incidences for ET, followed by PV and PMF [76]. A meta-analysis carried out on American and European studies mainly had the same distribution of the three diagnoses [3]. In Norway, a study conducted in 2017 with 2453 people showed that TE may have slightly higher prevalence than PV, and of the three, PMF had the lowest incidence [77].

On the other hand, there was high heterogeneity in the purposes of the included studies. One of the topics evaluated was epigenetic alterations. Studies on this topic have been very relevant to the explanation of pathogenesis and the evaluation of the influence of nongenetic factors on the development of MPN [31]. Currently, epigenetics is an important topic of study in the field of pharmacogenomics in many types of diseases, and through it we try to identify therapeutic targets for conventional therapy that are specific to each patient to encourage the application of personalized medicine [78]. In addition, studies that have focused on the hematological and clinical characteristics of the MPN have been very valuable because they mark the initial guidelines for the diagnosis of suspicious patients and have defined medical and therapeutic behaviors.

Despite the diversity of publications in the present review, the interest of some researchers in the exploration of MPN at the genetic level was clear, both via the search for new mutations and by determining the frequency and implications at the level of diagnosis, prognosis and pathogenesis of mutations already described. This interest can be attributed to the impact that the different genetic markers have had on the understanding of this group of diseases, their reclassification by being able to separate them from CML as an independent group [79], the therapeutic orientation to more specific and less invasive targets [80], and the improved management of the progression of the disease, among other factors.

The driver mutations, which include alterations in the *JAK2*, *MPL* and *CALR* genes, constitute a subject of great interest in this area. Their importance lies in the high frequency reported in ET, PV, and PMF, as well as their specificity for diagnosis. It is evident from this review that the main interest has been the frequency and clinical correlation of these three mutations. However, several studies evaluated other mutations in the TET2 gene related to the development of blast crisis and acute myeloid leukemia, which is currently an interesting line of research [81].

In addition to the genetic screening of these three driver mutations, several authors strongly recommend a search for a *BCR-ABL1* fusion gene as an initial step for the diagnosis of MPN insofar as it allows the exclusion of chronic myeloid leukemia, which is the most frequent entity of this group of neoplasms. The development of molecular tests in this area of hematology has allowed the implementation of diagnostic algorithms that lead to an accurate diagnosis in a logical order according to the frequency of each neoplasm [82].

The most frequently studied mutations were *JAK2*V617F, *MPL* and *CALR*. *JAK2V617F* presents more frequently in PV, whereas in ET and PMF, in the absence of *JAK2*, different mutations in the *CALR* and *MPL* genes are present in a large proportion of patients. These mutations are responsible for both the onset and the pathogenesis of the disease, and alter important cellular signaling pathways [83]. The *JAK2* gene codes for the Janus kinase 2 protein, which participates in the JAK-STAT signaling pathway that is important in cellular processes such as proliferation and differentiation. The main alteration is a change of amino acids that generates the *JAK2V617F* mutation. However, insertions and deletions in exon 12 result in other mutations in the *JAK2* gene that are less frequent [84, 85]. The *CALR* gene codes for the calreticulin protein, which functions in calcium homeostasis and as a chaperone [9]. The mechanism by which mutations in *CALR* neoplasically transform cells is not completely clear, but it has been described as being dependent on interaction with receptors such as *MPL* [78]. The main mutation described for the gene of the trompopoietin receptor is *MPLW515L*, which causes an amino acid substitution in the protein and results in constitutive activation of the JAK-STAT signaling pathway [8].

The meta-analysis showed that the probability of presenting *JAK2V617F* mutation in patients with PV is 3.0 times higher than for ET and 4.0 times higher than PMF. The probability of having the *MPL* mutation is 3.0 times higher in patients with PMF compared with ET, and there are no significant differences in the probability of having *CALR* between the two entities. In addition, in the analysis of subgroups for the frequency of the J*AK2V617F* mutation carried out in our study, a greater association was found in the frequency of the mutation with the WHO 2008 diagnostic criteria, which can be attributed to the greater specificity for the diagnosis of these neoplasms defined in this year [79].

These results confirm that the *JAK2V617F* mutation can constitute an important marker for the diagnosis of PV. The presence of this mutation has important clinical and prognostic implications, given that homozygous patients present a higher risk of splenomegaly and cardiovascular events. Specifically, in patients with PV, this marker is associated with higher levels of hemoglobin and neutrophil counts and fibrotic transformation [86].

The results of our meta-analysis of *MPL* revealed an increased risk of having the mutation in PMF with respect to ET. For some authors, this finding was associated with the prognosis of patients with ET, as they show greater progression to fibrosis, lower levels of hemoglobin and greater age. A similar pattern was seen for other authors in cases of ET with mutations in *MPL.* These might represent prefibrotic states of PMF [87–90]; however, studies on this topic are few and it is not possible to establish a relationship between the mutation and the prognosis.

On the other hand, the detection of mutations in the *JAK2* gene and in other genes is greatly influenced by the sensitivity and specificity of the detection technique used, as well as by the type and management of the sample. In this regard, all these details should be taken into account as recommended in the guide for the molecular diagnosis of myeloproliferative neoplasms in the United Kingdom in 2012 [82].

These findings, in conjunction with WHO recommendations, show that a diagnosis of MPN should not be isolated from genetic markers, and therefore, both the clinical and hematological characteristics and the presence or absence of these mutations should be taken into account. In addition, the findings show that mutations in exon 12 of the *JAK2* gene should also be considered before discarding PV [1].

Among the limitations of this study is the impossibility of explaining the sources of heterogeneity in the meta-analyses, as the individual studies were not exhaustive in their reporting of results by subgroups. The great advantage of this work is that it allowed the characterization of publications on MPN by general topics, such as years of publication, countries, and topics of interest, among others. Additionally, through this study, the current panorama of analytical investigations in this group of diseases was determined.

## Conclusion

The largest proportion of studies come from countries with high economic and research development and have been published mainly in the last 3 years. Despite the heterogeneity found in the purposes of the studies, there was a greater interest in genetic approaches, mainly concerning the *JAK2V617F, MPL* and *CALR* mutations. Given their specificity and high reported frequencies in this group of neoplasms, the diagnosis of these entities should not be made only by clinical and hematological characteristics alone but also by the genetic screening of patients. Finally, the presence of the driver mutations can be closely related to clinical and prognostic factors in this group of neoplasms; however, it is necessary to develop a greater number of studies on this topic, as well as the systematization of these studies to determine the real association of these frequencies with other aspects of the disease.

## Data Availability

All the data are available in the article.

## Abbreviations

CALR: Calrreticulin
CML: Chronic Myeloid Leukemia
ET: Essential Thrombocythemia
JAK2: Janus kinase 2
MPL: Trombopoietin receptor
MPN: MyeloProliferative Neoplasms
PMF: Primary MyeloFibrosis
PRISMA: Preferred Reporting Items for Systematic Reviews and Meta-analyses
PV: Polycythemia Vera
WHO: World Health Organization
